# Supplementary material for: Preoperative CT-Based Skeletal Muscle Mass Depletion and Outcomes after Total Laryngectomy
Source: Cancers (Basel). 2023 Jul 8;15(14):3538. doi: 10.3390/cancers15143538 (PMC10377557; doi:10.3390/cancers15143538)
Supplement: Supplementary file 1 [file cancers-15-03538-s001.zip › cancers-2436237-supplementary.pdf]

Supplementary Materials: Preoperative CT-based skeletal muscle mass depletion and outcomes after total laryngectomy

Victoria Salati, Katerina Mandralis, Fabio Becce, Joachim Koerfer, Karma Lambercy, Christian Simon and François Gorostidi

Table S1. Univariate and multivariate analyses of factors associated with all wound-related complications.

	Univariate		Multivariate	
	**OR (95% CI)**	**P-value**	**OR (95% CI)**	**P-value**
**Age**	0.97 (0.92-1.02)	0.206		
**Male gender**	1.06 (0.35-3.20)	0.916		
**BMI**	0.97 (0.90-1.04)	0.396		
**ASA ≥3**	1.33 (0.54-3.34)	0.537		
**Smoking**	1.52 (0.62-3.76)	0.359		
**SM depletion**	0.83 (0.35-1.98)	0.679		
**IMATI**	1.03 (0.93-1.16)	0.551		
**SMI**	0.98 (0.94-1.03)	0.462		
**SMRA**	1.00 (0.94-1.06)	0.962		
**Prior neck dissection**	0.26 (0.05-0.95)	0.056		
**Prior locoregional RT**	1.45 (0.61-3.52)	0.407		
**Prior chemotherapy**	1.99 (0.74-5.63)	0.178		
**Prior tracheostomy**	0.86 (0.29-2.54)	0.784		
**Flap reconstruction**	3.63 (1.50-9.20)	**0.005**	2.91 (1.14-7.65)	**0.027**
**Procedure**				
Total laryngectomy	-	-		
Pharyngolaryngectomy	2.92 (1.22-7.25)	**0.018**	2.16 (0.84-5.61)	0.109
**Neck dissection**	1.62 (0.26-12.79	0.609		
**Tumor location**				
Laryngeal	-	-		
Pharyngeal	2.16 (0.66-7.82	0.214		
Laryngo-pharyngeal	1.80 (0.57-6.03	0.321		
**Indication of surgery**				
Primary	-	-		
Salvage	3.89 (1.14-10.87)	**0.007**	3.64 (0.28-95.49)	0.350

* Significant p-values (<0.05) are displayed in bold characters.
